# Rethinking intraspecific diagnostics in *Lymantria dispar*: from traditional taxonomy to population genomics

**DOI:** 10.3389/finsc.2026.2009950

**Published:** 2026-10-29

**Authors:** Zarina Dairbekova, Bakyt Dulat, Alibek Makhambetov, Sofya Sviderskaya, Abay Sagitov, Dilyara Gritsenko

**Affiliations:** 1 Laboratory of Molecular Biology, Institute of Plant Biology and Biotechnology, Almaty, Kazakhstan; 2 Department of Molecular Biology and Genetics, Al Farabi Kazakh National University, Almaty, Kazakhstan; 3 Research Center AgriBioTech, Almaty, Kazakhstan

**Keywords:** Flighted Spongy Moth Complex (FSMC), introgression, *Lymantria dispar*, mitochondrial–nuclear discordance, molecular diagnostics, population genomics, taxonomy

## Abstract

The spongy moth, *Lymantria dispar*, provides a compelling example of how advances in genomic methods can reshape the practical interpretation of intraspecific taxonomy. Historically, geographic forms of this species were differentiated on the basis of morphology, geographic range, and female flight capability; subsequently, a three-subspecies framework comprising *L. d. dispar, L. d. asiatica*, and *L. d. japonica* became widely adopted. This classification has direct phytosanitary relevance because Asian forms are associated with a greater potential for dispersal and international transport. However, subspecies identity, geographic provenance, genomic ancestry, and traits associated with phytosanitary risk represent distinct types of information and require separate interpretation. Molecular studies have shown that diagnostic signals do not always coincide: RAPD, microsatellite, COI, and locus-specific markers can discriminate selected reference samples effectively, but their performance for ancestry inference and subspecies assignment depends on marker characteristics and reference coverage. Contemporary genome-wide SNP studies have identified broad patterns of genetic differentiation and admixture across Eurasia, including a pronounced Japanese component and a gradual transition between western Eurasian and East Asian populations. An additional layer of complexity is introduced by mitochondrial–nuclear discordance, whereby a maternally inherited mitochondrial lineage may not reflect an individual’s overall nuclear ancestry. This review evaluates the complementary roles of traditional taxonomy, early molecular markers, population genomics, and targeted SNP diagnostics in *L. dispar* identification and intraspecific characterization. We evaluate which practical aspects of diagnosis have been substantially improved, which limitations remain, and why future phytosanitary diagnostics may benefit from complementing categorical subspecies assignment with an integrative assessment of species identity, geographic provenance, genome-wide ancestry, while incorporating risk-associated traits remains a future objective requiring further validation.

## 1 Introduction

The spongy moth, *Lymantria dispar* (1), is a major defoliator native to Eurasia and northern Africa and established following introduction in parts of North America (2). Its phytosanitary significance reflects a broad larval host range, aerial dispersal of early-instar larvae, passive transport of egg masses on ships and cargo, and female flight capability in some populations (3, 4). Geographic variation in these traits makes intraspecific characterization relevant to biosurveillance. However, taxonomic identity, regulatory classification, geographic provenance, genomic ancestry, and traits contributing to invasion risk require separate interpretation.

Following Pogue and Schaefer (5), a widely used taxonomic framework recognizes *L. d. dispar, L. d. asiatica*, and *L. d. japonica*. Taxonomic classification assigns organisms to named taxa, whereas regulatory classification defines the units managed under a particular phytosanitary system. For example, the Flighted Spongy Moth Complex (FSMC) is an operational grouping that includes *L. d. asiatica* and *L. d. japonica* together with *L. albescens, L. umbrosa*, and *L. postalba* (6). It therefore extends beyond intraspecific classification within *L. dispar.* Subspecies designation has historically served as a proxy for geographic origin and risk, but it does not directly establish an individual’s source location, nuclear ancestry, or flight phenotype. Female flight capability varies geographically and within populations (3) (**Table 1**).

**Table 1:** Geographic overview of *Lymantria dispar* under the conventional three-subspecies framework.

Subspecies	Broad native distribution	Occurrence outside the native range	Key references
*L. d. dispar*	Europe, parts of western Asia, and northern Africa.	Established introduced populations in eastern North America, originating from Europe.	2, 5, 7, 8
*L. d. asiatica*	Primarily continental Asia east of the Ural Mountains, including Siberia, the Russian Far East, China, and the Korean Peninsula.	Included among Asian forms intercepted in North America and targeted by phytosanitary prevention and response programs.*	5, 8, 9
*L. d. japonica*	Japanese archipelago; the allocation of Hokkaido populations differs among taxonomic treatments.	Included among Asian forms intercepted in North America and targeted by phytosanitary prevention and response programs. *	5, 8–10

Native distributions summarize the conventional taxonomic framework and should not be interpreted as fixed boundaries between discrete nuclear genetic groups. Interception records indicate transport outside the native range but do not, by themselves, demonstrate population establishment. Reports concerning Asian forms may use broader operational categories; assignment to a particular subspecies therefore requires attention to the diagnostic evidence.

Population structure, admixture, clinal variation, and mitochondrial–nuclear discordance describe related but distinct aspects of genetic variation. Population structure concerns differences in genetic composition among populations; admixture concerns genetic contributions from differentiated populations; and clinal variation describes gradual geographic changes in allele frequencies or genetic affinities. Mitochondrial–nuclear discordance occurs when mitochondrial and nuclear data yield different patterns of genetic affinity or differentiation (8, 11). Such discordance requires attention to the nuclear markers examined: disagreement between mitochondrial DNA and a single nuclear locus does not, by itself, establish the extent of genome-wide admixture or introgression.

Range-wide genomic analyses have revealed a broad east–west genetic cline across continental Eurasia rather than a sharp nuclear genetic boundary between populations conventionally assigned to *L. d. dispar* and *L. d. asiatica*. In the same study, specimens sampled from Honshu and western Hokkaido showed separation from continental populations in principal component analysis and formed a distinct Japanese group in clustering analyses based on genome-wide nuclear SNPs (2). These findings characterize population differentiation within the sampled reference framework; they do not themselves constitute a formal taxonomic revision.

For this review, we distinguish four diagnostic objectives. Taxonomic identification establishes species identity and, where adequately supported, assignment to an intraspecific taxon. Geographic provenance inference evaluates a specimen’s likely source among the locations or populations represented in a reference dataset. Ancestry and admixture estimation assess nuclear genetic affinity and contributions from differentiated reference populations. Assessment of traits associated with phytosanitary risk evaluates phenotypes relevant to introduction, establishment, or spread, using direct observations or independently validated genotype–phenotype relationships. Geographic provenance and genomic ancestry are therefore distinct: genetic affinity to a reference population does not necessarily identify the location from which a specimen was transported.

We examine morphological, mitochondrial, locus-specific, multilocus, and genomic approaches in relation to these objectives. These methods provide complementary information and differ in inheritance, resolution, cost, scalability, and suitability for particular specimens and diagnostic questions. Genome-wide ancestry inference and reliable detection of a species or a particular allele impose different requirements, and performance in one task should not be generalized to another. We evaluate how the three-subspecies framework relates to observed genetic variation, how reference coverage and validation constrain diagnostic interpretation, and how methods can be combined according to the phytosanitary question. Genomic prediction of risk-associated phenotypes is considered a developing research direction requiring further validation, particularly for complex traits such as female flight capability (12).

### 1.1 Literature search strategy

This review was conducted as a structured narrative review rather than a systematic review. Relevant literature was identified through searches of Web of Science, Scopus, PubMed, and Google Scholar, with the final search conducted in September 2026. ResearchGate and publisher websites were additionally used to locate relevant publications and obtain full texts. No lower publication-date limit was applied because historical taxonomic literature and early molecular studies were required to reconstruct the development of intraspecific classification and diagnostic approaches in Lymantria dispar. Reference lists of key taxonomic, molecular-diagnostic, and population-genetic studies were also screened manually to identify additional relevant publications. References were organized using Zotero.

Searches used combinations of organism-specific terms, including *Lymantria dispar*, “spongy moth”, “Asian spongy moth”, “European spongy moth”, and “Flighted Spongy Moth Complex”, together with terms related to taxonomy and identification (taxonomy, subspecies, morphology), molecular diagnostics (RAPD, AFLP, microsatellite, COI, DNA barcoding, qPCR, TaqMan), genomics (SNP, GBS, population genetics, population genomics, genome-wide, assignment), and population processes (hybridization, admixture, introgression, and mitochondrial–nuclear discordance). Terms related to phytosanitary relevance, including quarantine, biosurveillance, and flight capability, were used where appropriate. Search terms were combined using Boolean operators according to the topic being investigated.

Peer-reviewed original research articles, review articles, and relevant taxonomic books or monographs were considered when they addressed the taxonomy, morphology, intraspecific identification, geographic differentiation, molecular diagnostics, population structure, population genomics, hybridization, or phytosanitary interpretation of *L. dispar*. Official phytosanitary and regulatory documents were additionally consulted when regulatory status or operational classification was discussed. Publications dealing exclusively with pest control, physiology, ecology, or other topics without relevance to the objectives of the review were excluded. Study selection emphasized publications that introduced or evaluated diagnostic methods, expanded geographic sampling, or provided new information relevant to the interpretation of mitochondrial, nuclear, genomic, or phenotypic variation. Because the aim of the review was a critical historical and biological synthesis rather than quantitative evidence pooling, no meta-analysis or formal risk-of-bias assessment was performed.

## 2 Biology, distribution, and phytosanitary significance of the spongy moth

### 2.1 Biological traits associated with invasion risk

Intraspecific variation in *L. dispar* is relevant to phytosanitary management because populations differ in traits influencing dispersal and host utilization (3, 13). Characterizing this variation can inform surveillance and risk assessment, although taxonomic identity and risk-associated phenotype require separate interpretation.

*Lymantria dispar* is univoltine and develops through egg, larval, pupal, and adult stages. It overwinters as diapausing eggs, with larvae hatching in spring and feeding before pupation and adult emergence. Adults do not feed; consequently, host utilization concerns the larval stage, whereas female flight and oviposition concern adults. Egg masses provide an important stage for passive transport, while newly hatched larvae can disperse aerially by ballooning (9, 14).

For identification and biosurveillance, the most relevant biological traits are those that affect the probability of transport, successful establishment, and subsequent spread. One such trait contributing primarily to establishment potential is the exceptionally broad host range of *L. dispar*. More than 500 plant species have been recorded as hosts of flight-capable Asian populations, while the species as a whole can exploit a wide range of woody plants (13, 15, 16). This broad host range should not be interpreted as a diagnostic characteristic of flight-capable populations or as evidence of a direct association between host breadth and female flight capability. The host spectrum is dominated by broadleaved plants, although some geographic populations can also develop on certain coniferous hosts (13, 17). Because host availability is one of the factors affecting establishment success, such broad polyphagy increases the likelihood that suitable food resources will be encountered following introduction into a new region (18).

A key biological trait historically used in phytosanitary risk assessment is the capacity of females for active flight (3). Comparison of 46 geographic strains showed that strong directed flight occurs in several populations from Asia, Siberia, and northeastern Europe, whereas no flight-capable females were detected among the North American, western European, or southern European populations examined (3). However, this phenotype is not fixed at the subspecies level or even within individual geographic populations: female flight capability varies clinally across Eurasia, and individuals with different degrees of flight capability can coexist within the same population (3, 19). Laboratory assessments distinguish sustained ascending flight, gliding, and flightlessness (3). For example, flight-mill tests reported mean accumulated distances of 2.64 ± 0.32 km for one-day-old females from Chinese populations (20). Such measurements describe experimental flight capacity rather than realized displacement in the field. Wing morphometrics correlate with flight capability at the population level; however, such correlations do not allow reliable inference of either the flight phenotype or geographic origin of an individual intercepted specimen, and morphological characters do not provide unequivocal identification of taxonomic status (3, 21, 22). Thus, female flight capability—a trait that has contributed substantially to the elevated regulatory concern associated with Asian forms—is not, by itself, a reliable marker of subspecies identity.

A second major component of phytosanitary risk is the transport of egg masses on ships and other artificial substrates. This invasion pathway involves several distinct biological and transport processes, including active female flight, attraction to artificial lighting, oviposition on artificial substrates, passive transport of egg masses, and subsequent larval dispersal. In East Asian port areas, flight-capable females are attracted to artificial lighting and may deposit viable egg masses on vessels, shipping containers, cargo, vehicles, and other components of port infrastructure (9, 23). These egg masses can remain viable during international maritime transport; after infested vessels arrive, emerging larvae can disperse aerially by ballooning and reach vegetation surrounding ports (4, 9, 24). The combination of active female flight, attraction to port lighting, oviposition on artificial substrates, long-distance passive transport of egg masses, and subsequent larval dispersal has created a distinctive pathway of international *L. dispar* introduction and provided the basis for multilayered vessel monitoring, inspection, and certification programs in both source and destination countries (9).

### 2.2 Taxonomy as an operational classification of risk

Phytosanitary regulation gives intraspecific identification of *L. dispar* direct practical significance. In the United States, *L. d. dispar* is a long-established invasive form, distributed mainly in the northeastern part of the country and regulated through federal and state quarantine measures intended to limit its further spread (25). By contrast, *L. d. asiatica* and *L. d. japonica* are included in the regulatory category known as the Flighted Spongy Moth Complex (FSMC), which APHIS treats as exotic to the United States; introductions are therefore subject to early detection and eradication measures (14, 16). The FSMC is a broader operational regulatory grouping that includes other *Lymantria* species in addition to *L. d. asiatica* and *L. d. japonica* and should not be interpreted as equivalent to the intraspecific taxonomic framework of *L. dispar*. Within the Eurasian Economic Union, *L. d. asiatica* is likewise regulated as a distinct quarantine pest (26, as amended). Consequently, the same species-level diagnosis of *L. dispar* may lead to fundamentally different regulatory decisions depending on the inferred geographic provenance or subspecific assignment of the specimen. Subspecies identity has historically been used in phytosanitary practice as a proxy for geographic origin and certain risk-associated traits, particularly female flight capability, rather than as a direct measure of an individual’s phytosanitary risk (3, 27). However, the relationship between subspecies identity and risk-associated phenotype is not absolute: female flight capability varies both among and within geographic strains, whereas genome-wide data reveal clinal structure and mixed ancestry in contact zones between *L. d. dispar* and *L. d. asiatica* (2, 3, 19, 28, 29).

For phytosanitary purposes, four related but distinct tasks can therefore be distinguished: establishing taxonomic identity, inferring likely geographic provenance, estimating genomic ancestry or admixture, and assessing traits directly associated with invasive potential (8, 30). These tasks provide different types of biological information and should not be treated as interchangeable.

Historically, these levels of information were often collapsed into the simpler dichotomy of European versus Asian spongy moths, reflected in diagnostic systems designed primarily to distinguish *L. d. dispar* from *L. d. asiatica*/*L. d. japonica* (27, 31). Introgression and discordance among mitochondrial, nuclear, and phenotypic signals highlight the limitations of applying such a binary model universally across the species range.

## 3 Limitations of traditional identification of *Lymantria dispar* subspecies

*Lymantria dispar* was described by Linnaeus (1), and subsequent examination of populations across its extensive Eurasian range resulted in the description of numerous geographically defined forms. Approximately 15 nominal subspecies have been assigned to *L. dispar*, reflecting variation in adult coloration and wing dimensions, larval patterning, geographic distribution, and other phenotypic traits (29, 32). Among the characters subsequently used to distinguish the major geographic forms were female forewing size, adult wing coloration and pattern, including the prominence of the postmedial band, and several larval characters (5). *L. d. japonica* was additionally characterized by comparatively large wings and a brownish cast to the female wings, whereas *L. d. asiatica* was distinguished from *L. d. dispar* partly by larger female wings and differences in adult coloration and larval patterning (5, 29).

The taxonomic interpretation of this variation has changed repeatedly. Schintlmeister (32) synonymized most previously described geographic forms and treated *L. d. asiatica* as a synonym of *L. d. dispar*, effectively recognizing *L. d. dispar* and *L. d. japonica* as the principal subspecific entities (32). Pogue and Schaefer (5) subsequently reinstated *L. d. asiatica* and formalized the three-subspecies framework—*L. d. dispar*, *L. d. asiatica*, and *L. d. japonica*—that became widely adopted in phytosanitary literature (5). This interpretation was not universally accepted: alternative classifications persisted, particularly for Japanese and Hokkaido populations, and later authors continued to question both the validity of *L. d. asiatica* and the delimitation of Japanese forms (29, 33). Thus, the validity and delimitation of *L. d. asiatica* have remained historically controversial, and the three-subspecies framework should not be interpreted as a definitive taxonomic resolution.

More recent taxonomic treatments have continued to recognize uncertainty within Japanese populations. In particular, the taxonomic status of Hokkaido populations has been interpreted differently among classification systems: *L. d. hokkaidoensis* has been recognized in some treatments, whereas other classifications have associated the name *hokkaidoensis* with *L. umbrosa*, and the applicability of the names *umbrosa* and *hokkaidoensis* remains debated (5, 7, 10).

Experimental crosses between Hokkaido and Honshu populations have also revealed reproductive incompatibilities, providing additional evidence of biological differentiation between these geographic forms (33). Nevertheless, this alternative treatment has not replaced the three-subspecies framework widely used in phytosanitary practice, illustrating the distinction between taxonomic hypotheses and operational regulatory classifications. Importantly, the characters used to support this framework are not individually diagnostic. In a comparison of 46 geographic strains, forewing dimensions generally followed the expected subspecific ranges but showed notable exceptions, while female wing coloration and markings did not accurately predict subspecies identity (3). The postmedial band proposed as characteristic of *L. d. asiatica* is likewise variable among Asian populations (3, 16), and the genitalia of the major subspecies are highly similar. Thus, traditional morphology primarily captures population-level tendencies rather than fixed diagnostic states.

The continued use of the three-subspecies framework may therefore reflect, at least in part, its operational usefulness in diagnostic and regulatory contexts. It provides a simple geographic classification that broadly corresponds to differences of phytosanitary importance, particularly the higher prevalence of female flight capability in Asian populations, and it became incorporated into molecular diagnostic and regulatory practice (9, 30). Its practical stability and adoption in regulatory practice, however, reflect operational utility and should not be interpreted as independent evidence that the three subspecies represent sharply delimited evolutionary lineages.

## 4 Development of molecular diagnostic methods for the *Lymantria dispar* complex

### 4.1 Early molecular diagnostics: searching for an “asian” genetic signal

The earliest molecular assays for *Lymantria dispar* were developed in response to a narrowly defined phytosanitary problem: distinguishing newly intercepted Asian moths from the established North American population of European origin (34–36). Under this application, mtDNA RFLPs, ITS2 polymorphisms, and RAPD-derived markers performed well because marker discovery and validation were based largely on geographically contrasting reference populations. Thus, these assays primarily provided geographic discrimination among strongly contrasting reference populations rather than taxonomic diagnosis across the full species range. Their apparent diagnostic specificity therefore likely reflected, at least in part, the strong differentiation between the populations used to develop the assays.

RAPD-PCR expanded the number of nuclear regions available for screening, but its usefulness for routine diagnostics was constrained by reproducibility and transferability. Garner and Slavicek (36) and Schreiber et al. (37) therefore converted informative RAPD fragments into sequence-characterized, locus-specific markers such as FS1 (36). This represented an important technical improvement because locus-specific PCR permits more stringent reaction conditions and is less sensitive to laboratory variation than anonymous RAPD fingerprints (37). Nevertheless, greater analytical reproducibility did not eliminate the underlying population-genetic limitation: a reproducible marker can still be geographically non-specific if the allele used for diagnosis also occurs outside the original reference populations.

This limitation was illustrated when the same marker systems were applied to substantially broader geographic sampling. Reineke and Zebitz (38) analyzed more than 1,000 moths from 18 European geographic sources and found RAPD profiles corresponding to patterns previously associated with Asian, North American, and hybrid reference samples at varying frequencies across most populations, whereas ITS2 classified none of the same European specimens as Asian (38). Thus, assays that had performed reliably in comparisons between Asian and North American reference samples were not directly transferable to European population diversity. The limitation was therefore not simply technical reproducibility, but reference dependence: marker specificity estimated from geographically extreme populations could not be assumed to apply across the continuous Eurasian range.

These early studies established a principle that remains relevant to current diagnostics. Validation against a small number of strongly differentiated populations can demonstrate analytical discrimination, but it does not necessarily demonstrate geographic specificity across the species range.

For phytosanitary use, marker validation should ideally include geographically intermediate and admixed populations, not only populations representing nominal subspecies extremes.

### 4.2 AFLP and microsatellites: from binary diagnostics to population assignment

AFLP and microsatellite markers reduced dependence on individual diagnostic loci by characterizing variation at multiple nuclear regions simultaneously. These approaches expanded the analysis of geographic genetic variation, while the scope of diagnostic interpretation remained dependent on reference sampling and validation. Reineke et al. (39), for example, analyzed 98 individuals from Europe, Asia, and North America using five AFLP primer combinations and detected extensive polymorphism and geographic clustering (39). However, much of the fine-scale evidence came from neighboring populations in southern Germany, while representation of the much larger Eurasian range was comparatively sparse. The study therefore demonstrated the sensitivity of AFLP for detecting geographic differentiation; however, it was not designed to establish or validate a universally transferable diagnostic classifier for unknown specimens.

AFLP generates multilocus fingerprints whose interpretation depends on standardized fragment scoring. Applying these profiles to routine regulatory identification would require validation across laboratories and geographically independent specimen collections. These operational requirements should be distinguished from the method’s demonstrated capacity to characterize population differentiation. The method generates large multilocus fingerprints, but the anonymous and predominantly dominant nature of AFLP markers complicates direct comparison among laboratories and makes assignment dependent on standardized scoring of electrophoretic profiles. In *L. dispar*, AFLP therefore proved particularly useful for population characterization and marker discovery, while being less readily adapted to simple routine identification of individual phytosanitary specimens.

Microsatellite analysis provided an important conceptual advance by enabling multilocus analysis of population structure and, when used within probabilistic assignment frameworks, statistical estimation of geographic affinity rather than inference from the presence or absence of a single putatively diagnostic allele. Genetic clustering characterizes patterns of differentiation within an analyzed dataset, whereas population assignment evaluates a specimen’s likely membership or source relative to defined reference populations. Bogdanowicz et al. (40) used only four microsatellite loci and four reference populations—Japan, the Russian Far East, China, and North America (40).

Assignment was strong for North American and Japanese moths, but some individuals from Russia and China did not assign correctly to their source populations. The authors explicitly cautioned that true Asian specimens could therefore appear North American at multiple loci, illustrating that probabilistic assignment does not remove reference bias when the underlying baseline is geographically incomplete.

A much broader analysis by Wu et al. (28), based on 1,738 individuals, nine microsatellite loci, and mitochondrial sequences spanning the Holarctic distribution, substantially improved geographic representation and revealed four major genetic clusters as well as extensive admixture in eastern and central Asia (28). This broader sampling demonstrated that, in contact zones, mixed ancestry may be a biologically meaningful result rather than an assignment failure, because admixed individuals may not correspond to a single discrete reference cluster. Thus, multilocus approaches represented a substantial improvement over binary diagnostic markers by allowing intermediate genetic affinity to be recognized rather than forcing all individuals into a single categorical group. For routine diagnostics, microsatellites are consequently more readily applicable to detailed population assignment than to rapid high-throughput screening, because they typically require genotyping and interpretation across multiple loci, whereas targeted qPCR assays can provide rapid detection of predefined variants with simpler analytical workflows (31, 41).

#### 4.3 COI barcoding and targeted qPCR: high practical utility, limited taxonomic depth

COI barcoding and targeted real-time PCR addressed several practical weaknesses of earlier fingerprinting methods. DNA barcoding provided a standardized sequence-based framework that could be compared across laboratories and reference databases, while TaqMan assays offered rapid, highly reproducible detection of predefined mitochondrial or nuclear variants. These properties facilitated the incorporation of COI barcoding and targeted qPCR into routine phytosanitary workflows for species identification and screening for predefined variants (30, 31, 41).

Islam et al. (41) evaluated the specificity, sensitivity, and repeatability of the FS1–18S multiplex assay using both laboratory-reared and field-collected moths (41). Stewart et al. (31) subsequently validated their multi-assay molecular key using 105 specimens and incorporated an internal logic for distinguishing multiple *Lymantria* taxa and for detecting the Asian-associated FS1 allele in moths carrying a *L. d. dispar* mitochondrial profile (31). Such assay architecture, together with rapid turnaround and compatibility with small or damaged specimens, makes qPCR particularly compatible with rapid routine screening workflows.

However, analytical validation must be distinguished from biological validation. Here, analytical validation refers to the technical performance of an assay in detecting its intended molecular target, whereas biological validation refers to the extent to which the detected marker reliably supports the intended biological classification across geographically representative populations. Accordingly, diagnostic sensitivity and specificity for geographic or subspecific classification depend not only on assay performance but also on the geographic representativeness of the validation dataset. COI represents a maternally inherited lineage, whereas FS1 represents a single nuclear locus. Consequently, neither marker alone provides a genome-wide estimate of ancestry. This distinction is especially important in Central Asia and other contact zones, where mitochondrial and nuclear signals can be discordant (11, 42).

Independent application of the Stewart et al. assay to natural populations from Kazakhstan illustrates this point. The assay produced clear but frequently discordant mitochondrial and FS1 marker signals (42). Because FS1 represents a single nuclear locus, this pattern should be interpreted as mitochondrial–nuclear marker discordance rather than as a direct estimate of genome-wide ancestry or the extent of introgression. This discordance does not, by itself, demonstrate poor analytical performance; rather, it highlights that mitochondrial and FS1 results describe different components of genetic variation and should not be interpreted as an unqualified subspecies assignment or a genome-wide ancestry estimate. Thus, targeted qPCR can reliably detect predefined genetic variants, but taxonomic inference from those variants may be limited when they are used as proxies for genome-wide ancestry.

The same issue applies to COI barcoding. A broad and well-curated reference library improves species-level identification, but subspecies-level inference remains vulnerable to mitochondrial introgression, incomplete lineage sorting, and geographic overlap of haplotypes. Accordingly, COI and targeted qPCR are most robust when used to answer narrowly defined questions—species identity, mitochondrial lineage, or presence of a particular nuclear allele—while their scope for inferring genome-wide ancestry or categorical subspecies assignment remains limited.

The development of microsatellite genotyping, COI barcoding, and targeted qPCR substantially improved reproducibility, throughput, and operational suitability. However, analytical performance and biological classification accuracy remain distinct properties: a marker can be detected with high technical reliability while supporting only a limited biological inference. This distinction helped motivate the subsequent shift toward genome-wide and population-based approaches.

## 5 Mitochondrial and nuclear genomic approaches to spongy moth identification

### 5.1 Mitochondrial phylogeography: high lineage resolution but limited inference of genome-wide ancestry

Beyond their use in routine identification, mitochondrial sequences have been used to investigate geographic differentiation within *L. dispar* and to identify lineage-informative variation at the whole-mitogenome level (11, 43, 44). Djoumad et al. (11) sequenced complete mitochondrial genomes from ten geographic variants of *L. dispar*, represented by one individual per sampled population, and identified a highly divergent mitochondrial genotype in a specimen from northern Iran. Additional COI–ND2 sequences from northern Iran and the Russian Caucasus showed close affinity to this genotype, suggesting the presence of a distinct regional mitochondrial lineage. Zhao et al. (22) subsequently reported related haplotypes from the broader Black Sea–Caspian region and proposed the provisional term “Black Sea–Caspian lineage” (22). These findings demonstrated that mitochondrial sequencing can reveal geographically structured variation not explicitly represented in the conventional three-subspecies framework. At the same time, the limited within-population sampling in early mitogenomic studies prevented reliable estimation of the frequency and geographic stability of such variants, and mitochondrial divergence alone should not be interpreted as evidence for formal recognition of a distinct subspecies or other taxonomic unit.

More fundamentally, increasing mitochondrial sequence length does not overcome the fact that the mitochondrial genome represents a single maternally inherited genealogy. Consequently, a complete mitogenome may provide high-resolution information on maternal lineage while remaining insufficient, on its own, for inference of biparental genome-wide ancestry (45).

Mitochondrial markers are therefore highly suitable for lineage identification, whereas inference of genome-wide ancestry requires nuclear population-genetic data, and any subsequent subspecies assignment represents a separate taxonomic interpretation of those data.

### 5.2 Nuclear reference genomes and genome-wide variation

The development of nuclear reference genomes substantially expanded the genomic resources available for *L. dispar*. Draft and long-read assemblies revealed a large, repeat-rich genome of approximately 0.9–1.0 Gb, and subsequent chromosome-level assemblies improved the framework for variant discovery and comparative analyses (46–50). However, a high-quality reference genome does not by itself provide a diagnostic system: reference assemblies generally represent one or a few individuals and therefore describe genomic structure rather than population-level allele frequencies. For geographic assignment, the critical resource is a sufficiently broad and representative population baseline. The broader genomic resolution provided by genome-wide markers is particularly valuable for estimating population structure and ancestry, but it should not be interpreted as inherently greater accuracy for taxonomic identification or phytosanitary risk assessment.

This distinction is illustrated by the first GBS-based assignment study. Picq et al. (51) obtained 2,327 SNPs from eight laboratory-reared populations and achieved 100% assignment of individuals to their source population (51). However, this accuracy was obtained within a closed set of laboratory colonies which may differ genetically from contemporary natural populations after laboratory maintenance. The authors explicitly cautioned that the resulting SNP panels were not immediately transferable to contemporary natural populations. The study should therefore be interpreted as a proof of concept demonstrating the potential resolving power of genome-wide SNPs rather than as validation of a field-ready diagnostic panel.

A broader field-based evaluation of transferability was provided by Wu et al. (8), who developed a 60-locus panel from more than one thousand field-collected moths sampled across Asia, Europe, North America, and North Africa (8). Unlike the earlier laboratory-based study, this panel was evaluated against natural population structure. Resampling of East Asian reference populations yielded an average DAPC assignment accuracy of 96.3%, while assignPOP cross-validation produced overall accuracies of approximately 93%. These values represent internal performance within the available reference framework and should not be interpreted as universal accuracy for specimens originating from populations not represented in that framework. Importantly, misclassifications were concentrated between genetically adjacent groups, particularly main China and the East Asian admixture zone, demonstrating that assignment accuracy decreases where reference populations are weakly differentiated or admixed (8). The use of two assignment algorithms provided an additional assessment of the consistency of geographic assignments.

Wu et al. (8) also applied the panel to intercepted egg masses and adults, providing an important demonstration of operational feasibility. However, the true geographic origins of intercepted specimens were generally not independently known; agreement between assignment methods therefore demonstrates analytical consistency rather than direct external validation of geographic provenance.

Thus, this approach represented a substantial advance toward routine biosurveillance but also illustrated a general limitation of population assignment: high cross-validation accuracy within a reference dataset does not guarantee equivalent performance for samples originating from unsampled, poorly represented, weakly differentiated, or admixed populations.

### 5.3 Range-wide population genomics and a test of the three-subspecies model

An important contribution of range-wide population genomics was not simply the increase in SNP number, but the expansion of the geographic reference baseline. Picq et al. (2) analyzed more than 2,000 GBS-derived SNPs in 1,445 contemporary moths sampled from 65 localities in 25 countries (2). This was substantially broader than earlier diagnostic datasets and recovered population differentiation at regional scales, including a pronounced Japanese component and a continuous genetic transition across continental Eurasia. The use of multiple analytical approaches also reduced dependence on a single clustering method and provided further evidence that the traditional three-subspecies framework does not map straightforwardly onto all components of the species’ genomic population structure.

The same range-wide analysis also revealed pronounced genomic differentiation of populations from the Caucasus and Middle East. Picq et al. (2) recovered these populations as a distinct genomic group and placed them as a sister lineage to *L. d. dispar* in a SNP-based phylogenetic analysis (2). Their genetic differentiation was sufficiently pronounced for the authors to suggest that this group warrants consideration as a separate subspecific unit. This does not constitute formal recognition of an additional subspecies, but it illustrates how genome-wide analyses can reveal differentiated regional units that are not represented explicitly in the conventional three-subspecies framework.

Nevertheless, “range-wide” sampling should not be interpreted as exhaustive sampling. The natural distribution of *L. dispar* extends continuously across much of Eurasia, whereas the genomic baseline consists of discrete collection localities separated in some regions by hundreds or thousands of kilometers. This limitation is particularly important in contact zones, where ancestry varies gradually rather than categorically. An unknown specimen originating between sampled localities can only be evaluated relative to the reference populations available in the model. Consequently, the precision of population assignment is influenced by how reference groups are geographically sampled and defined.

Increasing geographic resolution may therefore reveal additional ambiguity where adjacent populations are connected by continuous gene flow.

This consideration also affects interpretation of the three-subspecies model. A pronounced Japanese genetic component has been recovered consistently across several multilocus and genome-wide datasets, whereas the continental dispar–asiatica transition is less amenable to categorical subspecies assignment because geographically intermediate populations display admixture and mito-nuclear discordance (2, 8, 11, 28). The pronounced differentiation of the Caucasus/Middle East group further indicates that major regional genomic structure is not fully represented by the conventional three-subspecies classification. Genome-wide data therefore improve resolution of population structure and ancestry, but they do not necessarily produce sharper taxonomic boundaries or directly quantify phytosanitary risk. From a diagnostic perspective, an important advantage is their ability to quantify genetic affinity and mixed ancestry.

### 5.4 From genome-wide discovery to applied genomic diagnostics

One important obstacle to the routine use of genome-wide genotyping is that thousands of markers often provide more information than is required for operational biosurveillance while increasing cost, analytical complexity, and computational demands. A practical strategy is therefore to use range-wide genomic datasets for marker discovery and retain a smaller subset of loci that captures most of the information required for geographic assignments.

SpongySeq represents one of the most developed applications of this strategy currently available for *L. dispar*. Picq et al. (52) derived a 283-SNP AmpliSeq panel from a reference baseline comprising 1,156 moths collected from 61 sites in 25 countries and used it to distinguish 19 geographic reference groups. The panel was evaluated using known-origin adults and egg masses and was subsequently applied to intercepted egg masses from U.S. ports, providing a more operationally relevant assessment than earlier proof-of-concept SNP studies (52).

Nevertheless, validation of SpongySeq remains largely dependent on the reference collections and analytical framework used during its development. Broader independent evaluation across unrelated specimen collections, laboratories, sequencing platforms, and newly sampled populations is still required to determine the extent to which its assignment performance is transferable beyond the original reference baseline.

This distinction is important for routine diagnostics. A robust phytosanitary assay should ideally demonstrate not only high internal cross-validation accuracy but also interlaboratory repeatability, stability across DNA quality and life stages, portability among sequencing platforms, and accurate classification of geographically independent specimens. SpongySeq was developed on the Ion GeneStudio S5 platform, and the authors note ongoing efforts to adapt the assay to other sequencing technologies and to provide a user-friendly analytical interface (52). Such developments could facilitate broader adoption by improving both laboratory standardization and the consistency of assignment interpretation.

The progression from genome-wide discovery to reduced SNP panels represents an important step toward routine genomic biosurveillance. However, diagnostic performance should not be evaluated solely by the number of loci or by cross-validation accuracy within a predefined baseline. Continued expansion of geographically representative sampling, particularly in admixed transition zones, together with independent validation across populations and laboratories, remains essential. In this sense, genomic diagnostics do not eliminate reference dependence; rather, they make its importance more explicit.

## 6 Hybridization, introgression, and mitochondrial–nuclear discordance

Here, hybridization refers to interbreeding between genetically differentiated populations or lineages, whereas admixture describes the resulting pattern of mixed ancestry detected in individuals or populations. Introgression is used more specifically for the incorporation of genetic variants from one population into another through hybridization followed by subsequent backcrossing; therefore, evidence of admixture alone does not necessarily demonstrate introgression.

The genome-wide structure of *L. dispar* is most appropriately interpreted with consideration of historical and contemporary gene flow. Experimental crosses have long shown that geographic forms can produce viable offspring and subsequent backcross generations, although some combinations show asymmetric outcomes of reciprocal crosses and distorted sex ratios (19, 33). Thus, differentiation among forms does not imply complete reproductive isolation.

Multilocus and genome-wide studies have identified two prominent geographic regions showing mixed genetic ancestry. Across continental Eurasia, a broad dispar–asiatica transition extends from Eastern Europe through the Urals and Central Asia toward Siberia and East Asia; a second admixture zone connects continental East Asian and Japanese ancestry components in northeastern China, Korea, and the Russian Far East (2, 8, 28). In these regions, natural individuals display a broad continuum of mixed ancestry rather than a simple distinction between genetically “pure” and hybrid individuals. Such patterns may include reflect historical or contemporary gene flow, isolation by distance, repeated backcrossing, or other demographic processes, which cannot be distinguished from population-clustering patterns alone.

A particularly complex consequence is mitochondrial–nuclear discordance, in which the lineage or geographic affinity inferred from mtDNA does not coincide with the ancestry pattern inferred from nuclear markers. This pattern is widespread in animals and can arise through introgression, asymmetric gene flow, demographic processes, selection on mtDNA, or incomplete lineage sorting (45, 53). Discordance alone therefore does not establish a specific mechanism, but it precludes interpretation of any single genetic compartment as a complete characterization of an individual.

Central Asia provides a well-documented example of this pattern in *L. dispar*. Djoumad et al. (11) described individuals carrying dispar-like mtDNA but admixed nuclear characteristics, and Picq et al. (2) subsequently showed that such observations fit within a broad nuclear genomic cline (2, 11). A recent study of Kazakhstan provided a regional illustration of the same problem: all individuals showed the *L. d. dispar*-associated mitochondrial profile in the TaqMan assays, whereas the Asian-associated FS1 nuclear variant was detected in 92.8% individuals (42). These data are important not because FS1 provides an estimate of genome-wide ancestry or the extent of introgression—it represents only a single nuclear locus—but because they demonstrate mitochondrial–nuclear marker discordance and the risk of directly extrapolating COI identity to subspecies assignment.

The practical significance of introgression extends beyond the native range. Wu et al. (8) obtained evidence consistent with the occurrence of Asian–North American hybrids in the Pacific Northwest.

This observation raises the possibility that phytosanitary concern may extend beyond the establishment of introduced populations to subsequent gene flow and, potentially, introgression of particular alleles into established populations (8). Experimental crosses further showed that some flight capability can be retained in hybrid offspring, potentially affecting rates of spread (19).

However, it remains unclear which specific introgressed genomic regions directly modify invasion-relevant phenotypes (2, 51).

Mitochondrial–nuclear discordance should therefore be regarded not as a diagnostic defect, but as a biological property of the system (11, 45). COI can correctly identify the maternal lineage while remaining insufficient for inference of genome-wide ancestry (11); FS1 can correctly detect a specific nuclear allele while failing to characterize the proportion of Asian ancestry across the genome (51). This is a central lesson from *L. dispar* for molecular taxonomy: a technically correct marker result can still be overinterpreted taxonomically.

The major changes in the intraspecific classification of *L. dispar*, together with molecular and genomic findings that have revealed additional lineages and population structure, are summarized in **Table 2**.

**Table 2:** Historical taxonomic treatments and molecular findings in *Lymantria dispar*.

Period/source	Approach/evidence	Named taxa or taxonomic treatment	Genetic findings or operational groups	Interpretation
1	Morphology	*Lymantria dispar*	—	Original species description.
Subsequent taxonomic treatments	Morphology; geography	*Approximately 15 nominal subspecies*	—	Most were subsequently synonymized.
32	Morphology; geographic and type-locality data	*L. d. dispar; L. d. japonica*	—	*L. d. asiatica* was synonymized with *L. d. dispar.*
5	Adult and larval morphology; geography	*L. d. dispar; L. d. asiatica; L. d. japonica*	—	*L. d. asiatica* was reinstated, establishing the widely used three-subspecies framework.
54, as summarized by 10	Morphology; geographic distribution	*L. d. hokkaidoensis; L. d. japonica*	—	Alternative treatment of Japanese populations; the corresponding Hokkaido form was treated as *L. umbrosa* by Pogue and Schaefer (5).
28	Microsatellites and mtDNA	—	Four major nuclear genetic clusters	Population structure and admixture in eastern and central Asia; clustering does not constitute formal taxonomic recognition.
11, 22	Mitogenomes and mitochondrial markers	—	Black Sea–Caspian mitochondrial lineage	Regional maternal lineage; not formally recognized as a subspecies.
2	More than 2,000 genome-wide SNPs	—	Japanese genomic group; continental dispar–asiatica cline; Caucasus/Middle East genomic group	Genomic differentiation with possible taxonomic implications; no formal taxonomic revision was made.
52 — SpongySeq	Targeted 283-SNP panel	—	19 geographic reference groups used for assignment	Operational reference groups for provenance inference; not additional taxonomic units.

Taxonomic names reflect the classifications adopted in the cited historical treatments and do not imply universal acceptance. Genetic findings and operational groups are presented separately because mitochondrial lineages, nuclear clusters, genomic clines, and geographic assignment groups are not equivalent to formally named taxa. A dash indicates that the row does not summarize information of that type.

## 7 From traditional taxonomy to integrative genomic diagnostics

### 7.1 Problems that have been substantially improved

Over three decades of molecular diagnostics, several practical aspects of *L. dispar* identification and geographic assignment have been substantially improved. First, species identification no longer depends exclusively on diagnostic adult morphology: COI barcoding and PCR-based assays can be applied to eggs, larvae, and damaged or fragmented material (30, 31). Second, multilocus and genome-wide methods have enabled probabilistic assignment of unknown specimens to geographic reference groups (8, 40, 51, 52). However, the reliability of such assignments remains dependent on the geographic representativeness and completeness of the reference baseline. Third, subsequent population-genetic studies help explain why some early single-marker systems yielded apparently contradictory classifications: the population structure of *L. dispar* genuinely includes clines, admixture, and discordant mitochondrial and nuclear histories (2, 7, 8, 11).

At the conceptual level, genomics has also shown the question ‘Which subspecies is this?’ may not capture all information relevant to biosurveillance. For biosurveillance, the probability of geographic origin and the proportion of ancestry may provide information that is complementary to categorical subspecies assignment, while the future incorporation of validated risk-associated variants remains a longer-term objective (8, 12, 40, 52). SpongySeq represents a further practical step in geographic provenance assignment by translating genome-wide population structure into a manageable targeted panel for the analysis of intercepted material (52).

### 7.2 Remaining challenges

The first unresolved issue is the incomplete correspondence between the formal three-subspecies framework and the genomic population structure recovered in recent studies (2, 7, 8, 28, 29). Japanese *L. dispar* populations included in recent genomic datasets show comparatively consistent differentiation from continental populations, whereas the continental dispar–asiatica transition is more strongly characterized by clinal variation and admixture. These population-genomic patterns represent genetic structure rather than formal taxonomic revision, and the units recovered by such analyses should not automatically be interpreted as recognized subspecies. It remains unclear whether subspecies nomenclature should be revised, retained as a stable operational framework, or supplemented by a formal description of genomic ancestry. Any taxonomic revision should consider not only genetic distance, but also morphology, reproductive compatibility, ecology, and geographic history (2, 55).

The second challenge is incomplete reference sampling, particularly across geographically intermediate and admixed regions of Eurasia. Dense contemporary sampling is particularly important in Eastern Europe, the Caucasus, the Urals, Kazakhstan, Kyrgyzstan, western Siberia, northern China, and other transition zones, because these regions contain clinal variation, admixture, and additional intraspecific structure (2, 52). The completeness of the reference baseline is critical for assignment algorithms: an unknown specimen is evaluated only relative to the available reference groups, and if its true source population is absent from the baseline, it may be assigned incorrectly to the nearest represented group or remain without a confident assignment (52).

The third challenge is the imperfect and incompletely characterized relationship between taxonomy, genomic ancestry, and phenotypes directly relevant to phytosanitary risk. Female flight remains a central criterion of regulatory concern, but its genetic architecture is complex (3, 12). A recent integrative study combining genome-wide association analyses, whole-genome comparisons, and developmental transcriptomics identified numerous candidate genes and showed that flight capability is a multicomponent trait; a restricted set of candidates showed high classification performance, but the authors emphasized the need for additional validation of putatively causal flight-associated genes (12). The candidate gene sets also showed limited overlap among analytical approaches (12). Accordingly, individual candidate variants should not yet be treated as direct diagnostic markers of phytosanitary risk without further functional and population-level validation. Subspecies origin therefore remains an indirect proxy for flight-related risk (3, 12).

A further research direction is to investigate genomic variation beyond SNPs. Contemporary population-genomic and diagnostic panels for *L. dispar* are based predominantly on single-nucleotide variants (2, 51, 52), whereas structural variants, copy-number variation, and other classes of genomic variation remain much less studied at the population level. The continued development of long-read and chromosome-level assemblies for *L. dispar* provides the technical foundation for more comprehensive analysis of such variation (46, 48–50). In the longer term, population pangenomes may provide an opportunity to reduce reference bias and characterize structural variation that is poorly represented by standard SNP genotyping, although the diagnostic value of such approaches in *L. dispar* remains to be established (56).

### 7.3 A proposed integrative framework

For phytosanitary purposes, diagnostics can usefully be considered as a set of complementary components, the selection and combination of which should depend on the specific diagnostic question, specimen type, and available material. One component is confirmation of species identity using morphology and/or a standard DNA barcode (30, 31). The second is identification of the mitochondrial lineage as an independent characteristic, without automatically assigning the entire specimen to a subspecies (7, 11, 42). Multilocus nuclear assignment can be used with an appropriately validated population-genetic marker panel, such as a targeted SNP panel where a suitable reference baseline is available (8, 51, 52). Assessment of discordance and admixture may also be relevant: specimens showing conflicting mitochondrial and nuclear signals should be flagged as discordant and, where phytosanitarily relevant, subjected to broader nuclear ancestry analysis rather than being assigned solely from a single marker system (11, 42). As a future perspective, ancestry-based inference could eventually be complemented by validated information on phenotypes directly relevant to phytosanitary risk. This will require joint genotyping and standardized phenotyping of flight, fecundity, thermal tolerance, and other traits with direct relevance to establishment (3, 12, 19). However, many of these traits cannot be assessed directly in intercepted specimens, and their genetic determinants are not yet sufficiently validated for routine diagnostic use. Such a framework would separate two questions that traditional classification has often conflated: “Where does this individual originate?” and “What risk does it pose?” In the longer term, direct information on risk-associated phenotypes may provide an important complement to ancestry-based classification in phytosanitary risk assessment.

Finally, reference databases require greater standardization. They should also be continuously updated as new populations are sampled, expanded to improve geographic coverage, particularly in transition and admixture zones, and independently validated using specimens not included in the development of the original reference framework. Ideally, an internationally curated collection of contemporary voucher specimens should include verified geographic origin, morphology, mitochondrial haplotype, genome-wide nuclear genotype, and, where possible, phenotypic data (2, 30, 52). In contact zones, reference datasets should include not only “pure” geographic extremes but also naturally admixed populations (2, 52).

Where population-assignment methods are used, diagnostic reports could additionally provide probabilistic information, including nuclear assignment probabilities, evidence of admixture, and confidence levels (30, 52). Such a format would more explicitly represent the clinal population structure, admixture, and marker discordance documented in *L. dispar* than would a single binary label alone. The complementary roles of different diagnostic approaches are summarized in **Figure 1**.

**Figure 1:**
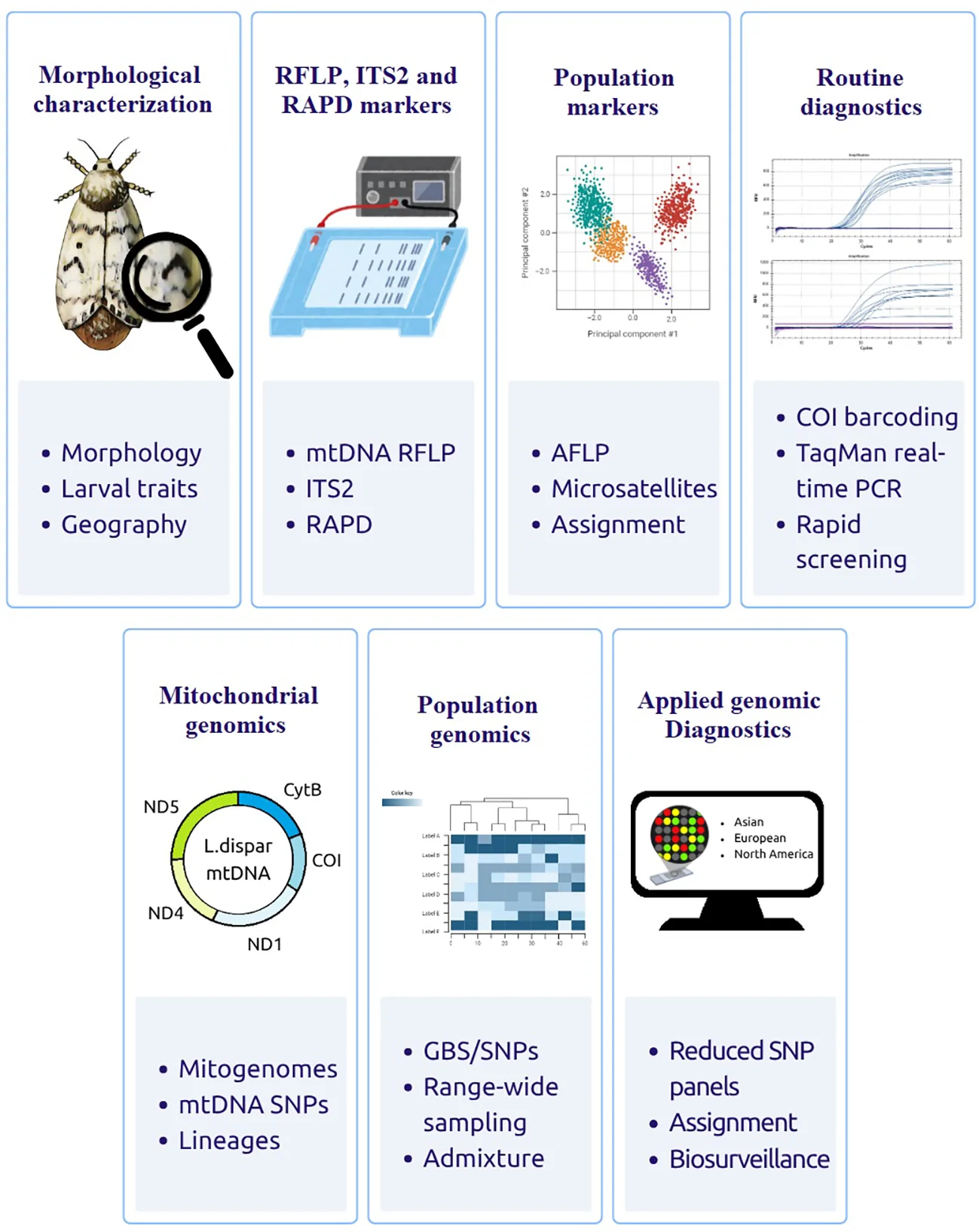
Complementary approaches for the identification and intraspecific characterization of *Lymantria dispar*. The approaches differ in the information they provide and are selected according to the diagnostic question, specimen characteristics, and available reference data.

## 8 Conclusions

The history of *L. dispar* demonstrates that the development of genomics has not simply increased the accuracy of an existing subspecies diagnosis; genomic approaches have enabled assessment of ancestry and population structure at a scale not accessible to traditional methods.

Morphology, geography, and female flight capability enabled the construction of a practical three-subspecies framework but did not provide a universal means of classifying individual moths. Early molecular markers helped address the practical problem of identifying life stages lacking diagnostic adult morphology and demonstrated heritable differentiation among populations, although their diagnostic specificity depended on the geographic composition of the reference samples.

Genome-wide data suggest that many difficult diagnostic cases may reflect underlying features of the species’ population structure, including broad genetic clines, admixture, and mitochondrial–nuclear discordance, rather than representing isolated exceptions. A single COI haplotype, the FS1 locus or geographic origin alone should therefore not be interpreted as an unconditional equivalent of subspecies identity.

Future methodological development may therefore benefit from integrative approaches that combine species identification, mitochondrial lineage, multilocus nuclear ancestry, and, in the future, sufficiently validated risk-associated traits, rather than relying on the expectation of a single universally diagnostic “Asian” marker. In this respect, the intraspecific variation observed in *L. dispar* illustrates a broader challenge in modern intraspecific taxonomy: discrete operational categories may intersect with continuous evolutionary and population variation, creating a need for diagnostic frameworks that represent this complexity without sacrificing practical applicability.
